# High-throughput microarray technology in diagnostics of enterobacteria based on genome-wide probe selection and regression analysis

**DOI:** 10.1186/1471-2164-11-591

**Published:** 2010-10-21

**Authors:** Torben Friedrich, Sven Rahmann, Wilfried Weigel, Wolfgang Rabsch, Angelika Fruth, Eliora Ron, Florian Gunzer, Thomas Dandekar, Jörg Hacker, Tobias Müller, Ulrich Dobrindt

**Affiliations:** 1 University of Würzburg, Institute for Molecular Infection Biology, Josef-Schneider-Str. 2/Bau D15, 97080 Würzburg, Germany; 2 Department of Bioinformatics, University of Würzburg, Am Hubland, 97074 Würzburg, Germany; 3 Bioinformatics for High-Throughput Technologies, Computer Science 11, TU Dortmund, 44221 Dortmund, Germany; 4 Scienion AG, Volmerstraße 7b, 12489 Berlin, Germany; 5 Robert Koch-Institute, Wernigerode Branch, Burgstrasse 37, 38855 Wernigerode, Germany; 6 Department of Molecular Microbiology and Biotechnology, Tel Aviv University, Tel Aviv 69978, Israel; 7 Faculty of Medicine Carl Gustav Carus, Institute for Medical Microbiology and Hygiene, Technology University (TU) Dresden, Dresden, Germany; 8 German Academy of Sciences Leopoldina, Emil-Abderhalden-Str. 37, 06108 Halle/Saale, Germany; 9 Institute for Hygiene, University of Münster, Robert-Koch-Str. 41, 48149 Münster, Germany

## Abstract

**Background:**

The *Enterobacteriaceae *comprise a large number of clinically relevant species with several individual subspecies. Overlapping virulence-associated gene pools and the high overall genome plasticity often interferes with correct enterobacterial strain typing and risk assessment. Array technology offers a fast, reproducible and standardisable means for bacterial typing and thus provides many advantages for bacterial diagnostics, risk assessment and surveillance. The development of highly discriminative broad-range microbial diagnostic microarrays remains a challenge, because of marked genome plasticity of many bacterial pathogens.

**Results:**

We developed a DNA microarray for strain typing and detection of major antimicrobial resistance genes of clinically relevant enterobacteria. For this purpose, we applied a global genome-wide probe selection strategy on 32 available complete enterobacterial genomes combined with a regression model for pathogen classification. The discriminative power of the probe set was further tested *in silico *on 15 additional complete enterobacterial genome sequences. DNA microarrays based on the selected probes were used to type 92 clinical enterobacterial isolates. Phenotypic tests confirmed the array-based typing results and corroborate that the selected probes allowed correct typing and prediction of major antibiotic resistances of clinically relevant *Enterobacteriaceae*, including the subspecies level, e.g. the reliable distinction of different *E. coli *pathotypes.

**Conclusions:**

Our results demonstrate that the global probe selection approach based on longest common factor statistics as well as the design of a DNA microarray with a restricted set of discriminative probes enables robust discrimination of different enterobacterial variants and represents a proof of concept that can be adopted for diagnostics of a wide range of microbial pathogens. Our approach circumvents misclassifications arising from the application of virulence markers, which are highly affected by horizontal gene transfer. Moreover, a broad range of pathogens have been covered by an efficient probe set size enabling the design of high-throughput diagnostics.

## Background

*Enterobacteriaceae *are frequent causes of human infectious diseases. Nevertheless, this family also comprises a broad variety of non-pathogenic and commensal variants. Furthermore, *E. coli *K-12 strains such as strain MG1655 are well-known model organisms in genetics and molecular biology. The family of *Enterobacteriaceae *comprises a multitude of pathogenic strains from the genera *Salmonella*, *Yersinia*, *Klebsiella *and *Escherichia*. The diversity in pathogenicity and related clinical symptoms has lead to the definition of a variety of intestinal and extraintestinal *E. coli *pathotypes [1]. The group of intestinal pathogenic *E. coli *(IPEC) includes five pathotypes causing diarrheal diseases with distinct features in pathogenesis: Enterohaemorrhagic *E. coli *(EHEC) cause diarrhoea and haemolytic uremic syndrome. Enteropathogenic *E. coli *(EPEC) are known for 'attaching and effacing' virulence causing diarrhoea predominantly in children. Enterotoxigenic *E. coli *(ETEC) cause watery diarrhoea with high incidence in developing countries. Enteroaggregative *E. coli *(EAEC) have been frequently isolated from children and adults showing persistent diarrhoea. Host cell invasion characterises enteroinvasive *E. coli *(EIEC) which cause watery diarrhoea. EIEC are highly similar to *Shigella *isolates, which are clinically associated with varying degrees of dysentery. Furthermore various types of so-called extraintestinal pathogenic *E. coli *(ExPEC) have been described to cause infections outside of the gastrointestinal tract, i.e. urinary tract infection, newborn meningitis or sepsis. Generally, uropathogenic *E. coli *(UPEC), newborn meningitis-associated *E. coli *(MNEC) as well as sepsis-associated *E. coli *(SEPEC) differ in their repertoire of virulence-associated genes from IPEC [1].

*Salmonella enterica *infections can result in enteric fever caused by typhoid serovars (Typhi and Paratyphi) or gastroenteritis due to infection with the non-typhoid serovars (Typhimurium and Enteriditis) [2]. The genus *Yersinia *harbours three pathogenic species associated with plague (*Y. pestis*) and yersiniosis (*Y. enterocolitica *and *Y. pseudotuberculosis*) [3]. *K. pneumoniae *is predominantly isolated from patients with pneumonia or urinary tract infection and is, together with *E. coli *variants, frequently isolated from patients suffering from nosocomial infections [4]. Several characteristic virulence-associated determinants have been described for different enterobacterial pathogens [4-6].

Many enterobacterial sequencing projects have been finished so far and even more are in progress as part of comparative studies. The availability of increasing numbers of genomic sequences enables the development of new diagnostic strategies and further sequencing projects will improve and robustify these diagnostics. In the past, many studies have focused on the development of diagnostics mainly for single enterobacterial clades. Conventionally, such tests were based on PCR or multiplex PCR to detect variation in partial sequences of marker gene loci like 16S rRNA [7,8]. The development of the microarray technology enabled parallel investigation of multiple determinants while ensuring high reproducibility, thus facilitating high-throughput diagnostics [9].

Microbial diagnostic microarrays for the detection of pathogenic *E. coli *were designed based on polymorphisms in single genes [10,11] or on libraries of virulence determinants [12-14]. Moreover, the application of microarrays in antimicrobial resistance (AMR) screening [14-16] has implications on medical therapy and epidemiological studies [17,18]. However, a diagnostic microarray that allows rapid discrimination between different genera, species and even subspecies of clinically relevant enterobacteria has not yet been reported.

Here, the development of a microarray for high-throughput diagnostics of enterobacteria is described, which targets the identification of clinically relevant pathogroups from genus to even subspecies level. In contrast to previous work, we unravelled new pathogroup-specific capture probes by probe selection across whole groups of genomes. Our results reveal that multi-genomic probe selection also indicates the integrity of considered bacterial groupings. Diagnostic classification as well as the quantification of pathogens in a sample is provided by the application of a new regression model. The classifier features the adaptation of hybridisation data and thus constantly improves its classification.

## Results

### Concept of microarray design

**Figure 1:**
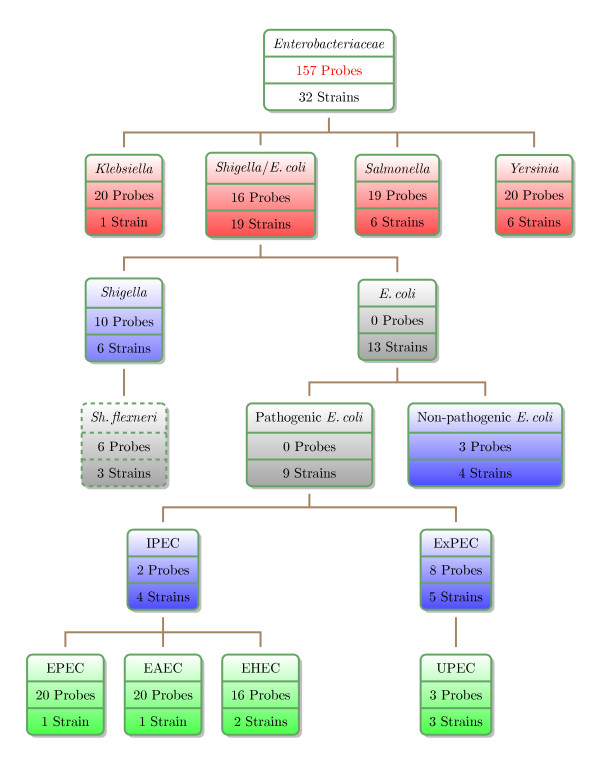
**Overview of assigned clinically relevant *Enterobacteriaceae***. Each node corresponds to a pathogroup entity and the respective box comprises information about the number of probes designed for the respective group as well as the number of strains assigned to the group according to prior knowledge. The colours refer to the genus level (red), the intermediate *E. coli *level (blue) and the *E. coli *pathotype level (green). Gray colour refers to pathogroups for which no probes could be found and the white box titled '*Enterobacteriaceae' *summarises the assignment.

Our strategy to design a diagnostic microarray based on a new set of pathogroup-specific determinants is structured according to clinically distinct enterobacterial pathogroups. Figure 1 depicts these subdivisions assigned to the *Enterobacteriaceae *and illustrates the nested relations associated with the versatile group of *Shigella *and *E. coli *strains. The hierarchical dendrogram is further denoted as the pathogroup tree.

The subdivisions applied in this case were guided by clinical relevance. The comparisons were split into three main levels of organisation within the pathogroup tree: (I) the genus level, (II) the distinction between *Shigella*, pathogenic and non-pathogenic *E. coli *as well as (III) the diversity among intestinal and extraintestinal *E. coli *pathotypes. The groups of *Shigella *and non-pathogenic *E. coli *were also contrasted to the pathotype level - a clinically reasonable differentiation. These splits have been included to avoid nested relations of pathogroups, which can lead to inaccuracies in classifications by regression analysis.

### Probe selection

The sequences of complete enterobacterial genomes, including plasmids, of reference strains (see part A of Table 1) were subjected to a probe selection procedure to find capture probes that provide a high discrimination capacity between the different levels of the pathogroup tree. The strategy of probe selection was based on a global extraction of group-specific 70-mer oligonucleotides by the application of longest common factor statistics [19]. Long probes as chosen here provide the advantages of reduced cross-hybridisation events and less chemical influence of the microarray surface on the hybridisation. Moreover, the outcome of provisional probes in the probe selection process (see below) provides a good coverage of all diagnostic groups. The string matching algorithm yielded sets of fully conserved oligonucleotides, which meet the criteria of valid capture probes as stated in the methods section, within a primarily unrestricted composition of genomic groups. Manual selection of probes from the provisional probe set (~18,000 oligonucleotides) was guided by clinical importance of enterobacterial subgroups (see Figure 1). The yield of provisional probes could be vastly enlarged by slight relaxation of selection criteria to ~360,000 probes (see methods section for details), but the large 'stringent set' put the need for additional probes aside. The set of candidate probes was carefully selected from the pool of provisionals according to cross-matching behaviour to human DNA and conventional hybridisation parameters (GC-content, melting temperature, change in Gibb's free energy, complexity in base composition).

**Table 1:** Enterobacterial Genome Sequences

Genus	Species	Isolate	Patho-/Serotype	Genbank-ID	Reference
*Part A - Reference genomes*					

*Escherichia*	*coli*	K-12 MG1655	non-pathogenic	U00096.2	[70]
		K-12 W3110	non-pathogenic	AP009048.1	[71]
		Nissle 1917	commensal	--	--
		O9 HS	commensal	CP000802.1	[35]
		536	UPEC	CP000247.1	[72]
		UTI89	UPEC	CP000243.1	[73]
		CFTO73	UPEC	AE014075.1	[74]
		O157:H7 EDL933	EHEC	AE005174.2	[75]
		O157:H7 Sakai	EHEC	BA000007.2	[76]
		O42	EAEC	N554766	[77]
		E2348-69	EPEC	FM180568	[78]
		
		APEC O1	APEC	CP000468.1	[79]
		
		789	APEC	--	--

*Shigella*	*flexneri*	2a 301	2a	AE005674.1	[80]
		5b 8401	5b	CP000266.1	[81]
		2a 2457T	2a	AE014073.1	[82]
	
	*dysenteriae*	Sd197	1	CP000034.1	[83]
	
	*sonnei*	Ss046	1	CP000038.1	[83]
	
	*boydii*	Sb227	4	CP000036.1	[83]

*Klebsiella*	*pneumoniae*	MGH78578		CP000647.1	[84]

*Salmonella*	*enterica*	Paratyphi ATCC9150	A	CP000026.1	[85]
		Choleraesuis SC-B57	C1	AE017220.1	[86]
		Typhi Ty2	D1	AE014613.1	[87]
		Typhi CT18	D1	AL513382.1	[88]
	
	*typhimurium*	LT2	B	AE006468.1	[89]
	
	*bongori*	12419	--	--	Sanger Institute

*Yersinia*	*pestis*	CO92	Orientalis	AL590842.1	[90]
		KIM	Medievalis	AE009952.1	[91]
		91001	Microtus	AE017042.1	[92]
		Antiqua	Antiqua	CP000308.1	[93]
		Nepal516	--	CP000305.1	[93]
	
	*pseudotuberculosis*	IP32953	--	CP000720.1	[33]
	
	*enterocolitica*	8081	--	AM286415.1	[94]

*Part B - Recently published genomes*					

*Escherichia*	*coli*	DH10B	non-pathogenic	CP000948.1	[95]
		ED1a	non-pathogenic	CU928162.2	Genoscope C.E.A.
		SE11	non-pathogenic	AP009240.1	[96]
		ATCC8739	non-pathogenic	CP000946.1	Joint Genome Institute
		IAI1	non-pathogenic	CU928160.2	Genoscope C.E.A.
		IAI39	UPEC	CU928164.2	Genoscope C.E.A.
		UMN026	UPEC	CU928163.2	Genoscope C.E.A.
		SMS-3-5	non-pathogenic	CP000970.1	[35]
		O157:H7 EC4115	EHEC	CP001164.1	J. Craig Venter Institute
		O157:H7 EC4115	EHEC	CP001164.1	J. Craig Venter Institute
		55989	EAEC	CU928145.2	Genoscope C.E.A.
		E24377A	ETEC	CP000800.1	The Institute for Genomic Research
		S88	MNEC	CU928161.2	Genoscope C.E.A.

*Shigella*	*boydii*	CDC 3083-94	18	CP001063.1	J. Craig Venter Institute

*Salmonella*	*enterica*	Enteritidis P125109	PT4	AM933172.1	[97]

*Klebsiella*	*pneumoniae*	342		CP000964.1	[31]

The chosen probe length has been described before as an optimal compromise between sensitivity and specificity [20]. Due to the objective to construct a slim and cost efficient diagnostic tool we restricted the size of the probe set to a maximum of 20 capture probes per pathogroup. Despite the large size of the provisional probe set, no pathogroup determinant could be defined for the generic entities '*E. coli*' and 'pathogenic *E. coli*' implicating the absence of concise genotypes across the respective strains. Similarly, the selection of probes mainly for the discrimination at the *E. coli *pathotype level did not always utilise the maximum number of capture probe, which was limited to 20 probes per pathogroup to guarantee a cost-efficient microarray architecture. Figure 1 details the number of capture probes assigned to each pathogroup. The topmost node entitled '*Enterobacteriaceae*' does not characterise a pathogroup but provides a summary of probe selection, which resulted in a probe set of 157 capture probes derived from 32 reference genomes. The probe set has been made publicly available [NCBI Probe Database puids: 10316816 to 10317025]. A detailed mapping of NCBI Probe Database Ids to the probes is given in additional file 1.

Due to the limited availability of bacterial genome sequence data, certain assigned pathogroups like EAEC or EPEC were underrepresented at the time of chip design (06/2006). Moreover, no genome sequences were publicly available at that time for the *E. coli *pathotypes ETEC, EIEC, SEPEC and MNEC. To compensate for individual unavailability of genomic data, comprehensive test hybridisations with bacterial DNA from a broad variety of strains were conducted to verify the discriminative power of the chosen capture probes.

By means of initially unrestricted group-wise probe selection we could specify probes separating *S. flexneri *as a *Shigella *subgroup (dashed box in Figure 1), though no special emphasis was put on such a subdivision. Since *S. flexneri *causes basically the same clinical symptoms as other *Shigella *species, the subgroup was not separately analysed.

### Characterisation of capture probes

Selected oligonucleotide probes were mapped to the genomes of respective groups by a BLAST search to find general annotations of corresponding group-specific, genomic regions. The annotations were summarised to the categories listed in Table 2 as column labels. In accordance to the applied generalised probe selection strategy, nearly 13% of probes originated from intergenic regions. Capture probes that could be associated to known genes cover a wide range of functional groups. Interestingly, the majority of selected probes refers to genes with poor or missing annotation (Table 2).

**Table 2:** Overview of oligonucleotide markers for pathotyping and their categorisation.

Group	Intergenic	Virulence	Uncharacterised	Transcription	Adhesion	Extracellular	Metabolism	Transport	Miscellaneous	Probeset
*Yersinia*	4	0	11	0	0	0	4	0	1	20
*Klebsiella*	7	0	5	1	1	0	4	2	0	20
*Salmonella*	1	0	6	0	2	0	4	3	3	19
*Shigella*/*E. coli*	1	0	3	4	0	1	7	0	0	16
*Shigella*	1	4	0	0	1	0	0	0	4	10
Non- pathogenic	1	0	0	0	0	0	1	1	0	3
ExPEC	0	0	4	0	0	2	2	0	0	8
UPEC	0	0	2	0	1	0	0	0	0	3
IPEC	0	1	1	0	0	0	0	0	0	2
EHEC	4	0	10	0	1	1	0	0	0	16
EPEC	0	0	16	1	1	0	1	0	1	20
EAEC	1	0	17	0	1	0	0	0	1	20
In total	20	5	75	6	7	5	23	6	10	157

The term "Probe set" refers to the contribution of genomic groups to the final set of probes. Beside several probes in categories like virulence, extracellular (secreted proteins) or transcription, the probe set comprises a relatively large fraction of probes originating from intergenic regions.

### Assessment of single probe performance

**Figure 2:**
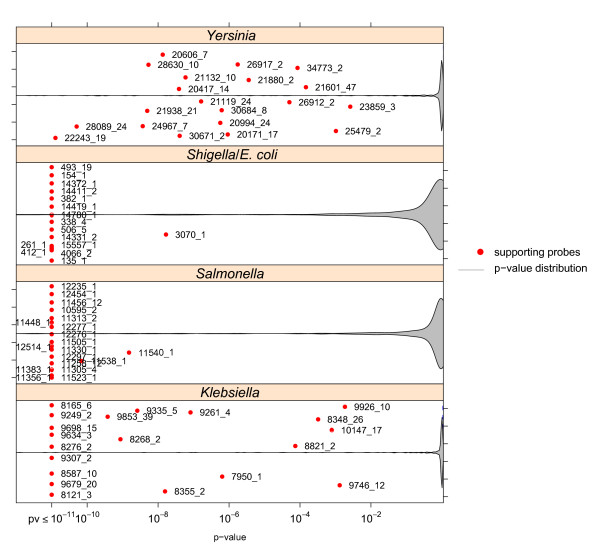
**Probe-specific contribution to the detection of diagnostic groups in the genus level of the pathogroup tree**. The performance of single group-specific probes in the detection of pathogroups was determined by a combination of an ANOVA and simultaneous inference of one-sided multiple comparisons. The resulting adjusted p-values communicate the robustness of intensity difference between pairs of pathogroups. The p-values from single comparisons were averaged for each pathogroup in log space. Violin plots indicate the overall distribution of non-averaged p-values on a log-scaled x-axis as relative densities (density values are not reflected by the y-axis). The y-axis follows arbitrary units in order to improve readability of single points.

**Figure 3:**
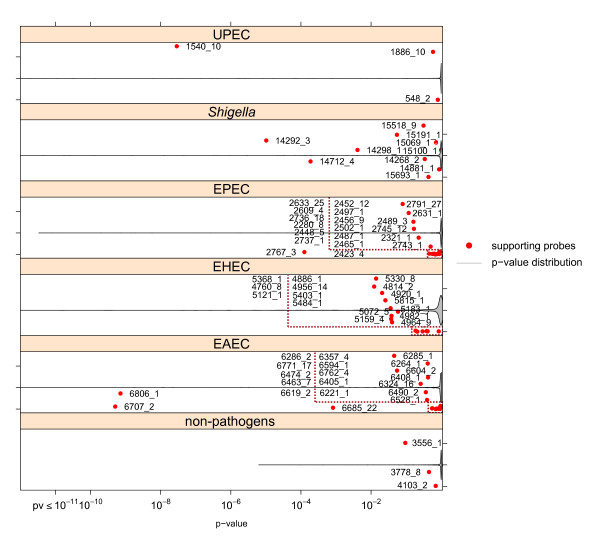
**Discriminative power of group-specific probes among *E. coli *pathotypes**. The ability of probe-wise discrimination derived from ANOVA-based simultaneous inference of one-sided multiple comparisons further decreases in the *E. coli *pathotype level of the pathogroup tree. Single red dots mark averaged adjusted p-values of group-specific probes. The overall distribution of p-values as indicated by violin plots exhibits a general increase of p-values. The increase is influenced by a larger number of comparisons and by the close relation of the target groups.

Comprehensive test hybridisations gave insights into the reliability of single group-specific capture probes in the classification of respective pathogroups. The significance of probe-specific contribution in group separation was determined by an analysis of variance (ANOVA) on signal intensities in conjunction with the method of simultaneous inference for parametric models [21] as post-hoc test. While the ANOVA yields the probability that the distributions of signal intensities do not exhibit any difference in mean, the method of Hothorn *et al. *determines adjusted p-values of individual differences in the mean concerning all pairwise one-sided comparisons between pathogroups in reference to a pre-specified maximum significance level. Resulting p-values for specific probes (represented as red circles in the plots) of each pathogroup against all others were averaged in log space to obtain capture probe-specific single indicators of pathogroup support. The averaged p-values of probes in respective groups are contrasted in Figures 2 to 3 against p-value distributions (not averaged) of differences in the mean signal intensity over all pairwise tests of a respective pathogroup and any capture probe. The p-value distributions are visualised as arbitrarily scaled densities of so-called violin plots on a log-scaled p-value axis (x-axis), which is cut at a p-value of 10^-11^. Low averaged p-values reflect a significantly higher mean signal intensity of a capture probe in its target pathogroup than in other pathogroups. Group specific probes that form the body of overall lowest p-values therefore highlight a success of probe selection.

As revealed by Figure 2, p-values of probes specific to genus-level pathogroups generally indicate high significance in the ability to classify respective strains. In comparison, the genera exhibit differences in the overall performance of corresponding probes. Best overall support was obtained for the '*Salmonella*' and '*E. coli*' pathogroups while lower but still clearly significant p-values were assigned to probes selected from *Klebsiella *and *Yersinia *genomes. These results seem to arise from quite different influences. The probes of the '*E. coli*' group were selected against the background of numerous genomic sequences which confer probe robustness. In contrast, the observed larger variability in '*Klebsiella*' probe performance reflects limited genomic data available in this group. '*Salmonella*' constitutes a pathogroup with a largely homogenous genotype [22]. '*Yersinia*' probe variability seems to mirror the genotypic diversity among *Yersinia *ssp. strains [23].

Figure 3 reflects averaged p-values of single capture probe performance in terminal pathogroups of the '*E. coli*' group. Group-specific capture probes again constitute the lowest fraction of the overall p-value distribution. The evaluation of '*Shigella*'-specific determinants resulted in four capture probes classifying all *Shigella *strains as well as those specific only for *S. flexneri*. The corresponding plot reveals significant support by the capture probes representing the whole group. The three top-performing '*Shigella*'-specific probes originate from the locus of the invasion plasmid antigen H gene (*ipaH*). The identification of the EAEC pathotype is strongly supported by two probes. One of these high-performing probes with the ID 6806_1 is located in the plasmid-encoded *aatD *gene locus.

Further information on the assessment of probe performance in the intermediate pathogroup level is given in additional file 2.

### Classification of hybridisation patterns

The global aim of any diagnostic means is the distinction between and the detection of targets, here clinically relevant enterobacterial pathogroups and antimicrobial resistance determinants, respectively. In the following, the ability of the developed microarray to come to such classifications is described. Comprehensive test hybridisations provide the basis for these investigations.

### Regression analysis

In order to predict the allocation to a diagnostically relevant enterobacterial pathogroup (see Figure 1), a regression model was trained with the results from test hybridisations analogous to recently described methods [24,25]. The regression model treats intensities of single probes independently from one another because of probe specific hybridisation behaviour. The target affinity to perfect match probes is dependent on the probe-specific sequence composition and does not allow for direct comparison of intensities from hybridisations to different probes. Given the intensity matrix of hybridisations *Y *with probes as rows and samples as columns as well as a master table *X *containing hybridised amounts of DNA of the same size, the linear regression model equates to

Y=AX

The affinity matrix is estimated by solving the equation

$$\hat{A}=YX^{T}{\left(XX^{T}\right)}^{-1}$$

The prediction performance of the regression model was determined by leave-one-out cross-validation: in a recurrent sampling procedure the regression model was trained in each run by all but a single hybridisation pattern, which further on served as test pattern. Based on the test pattern the amount of corresponding genomic DNA (gDNA) *x_k _*was predicted to

$${\hat{x}}_{k}={\hat{A}}^{-1}y_{k}$$

with *y_k _*being the intensity vector of test sample *k *and ${\hat{x}}_{k}$ representing a vector of predicted gDNA ratios of capture probes representing all incorporated pathogroups. Based on prior knowledge on the true nature of test strains a master table *X *was generated, which refers to the hybridised amount of DNA in each pathogroup. All capture probes characterising a certain pathogroup or its parent group of a test strain were set to an appropriate factor of hybridised DNA (for pure cultures 1.0 = 2 μg), while 0.0 was assigned to all other probes. The factor corresponds to the proportion of the sample DNA coming from a certain pathogroup and drops only below one in mixed culture hybridisations. Predicted amounts of hybridised DNA for single probes are mapped back to the pathogroup by taking the median of all pathogroup-specific probes. Each pathogroup was evaluated by samples from different strains. Groups with no explicit representations in the probe set (pathogroups without available reference genomes like ETEC, EIEC and SEPEC) were treated separately. In these cases, the amount of hybridised DNA was determined by a regression model estimated on all reference *E. coli *pathotypes.

### Pure cultures

**Figure 4:**
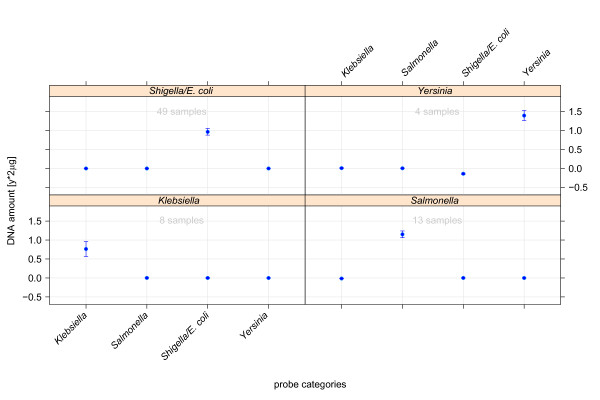
**Evaluation of the prediction performance on the genus level of the decision tree**. The four plots summarise the classifications on the genus level of enterobacteria subdivided into prediction outcomes of the pathogroups '*Shigella/E. coli*' (top left), '*Yersinia*' (top right), '*Klebsiella*' (bottom left) and '*Salmonella*' (bottom right). The headline of each plot refers to the true nature of the respective test samples and the x-axis represents the pathogroups, which are contrasted in regression model analysis. In this case the linear regression model was trained on signal intensities of probes representing the main genera of considered *Enterobacteriaceae *(*Salmonella*, *Shigella*/*E. coli*, *Klebsiella *or *Yersinia*, the x-axis). The model was trained with all hybridisation patterns. The medians with standard error of predicted DNA amounts were obtained by leaf-one-out cross-validation.

The regression model-based cross-validation has been determined in the context of the previously denoted intrinsic levels of the pathogroup tree. At the genus level (see Figure 4), all samples were classified correctly during cross-validation. Moreover, the regression model exhibited the ability to accurately predict DNA amounts used for hybridisation. The tests furthermore suggested an influence of sample coverage in the accuracy of quantitative predictions.

**Figure 5:**
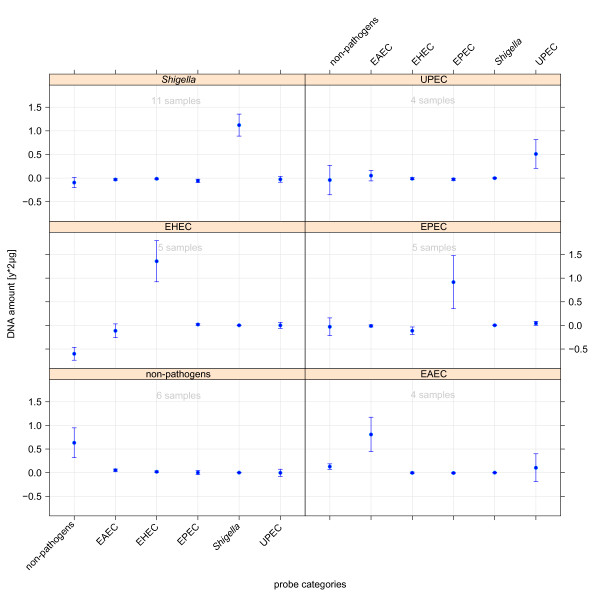
**The prediction of hybridised DNA of the groups beneath the node of *E. coli *and *Shigella *isolates**. The plot shows cross-validation results obtained by a regression model, which was trained only on signal intensities of probes associated to contrasted groups (x-axis). Filled circles indicate the predicted ratios of DNA in test samples of respective groups and the error bar indicates the cross-validation error of each prediction. The contrasted pathogroups comprise all integrated *E. coli *pathotypes as well as *Shigella *and non-pathogenic *E. coli*.

*E. coli *pathotypes exhibited a close phylogenetic relationship with largely collinear genotypes and high frequency of genetic interchange. For these low level pathogroups only few reference genomes were generally available per group. Therefore, the classification of *E. coli *pathotypes depicted in Figure 5 constituted the most difficult classification scenario within the presented setting. In the context of clinical relevance, *Shigella *and non-pathogenic *E. coli *pathogroups were included into this classification setting. In all classifications, the prediction level of the true class can be robustly separated from prediction levels of respective negative classes.

**Figure 6:**
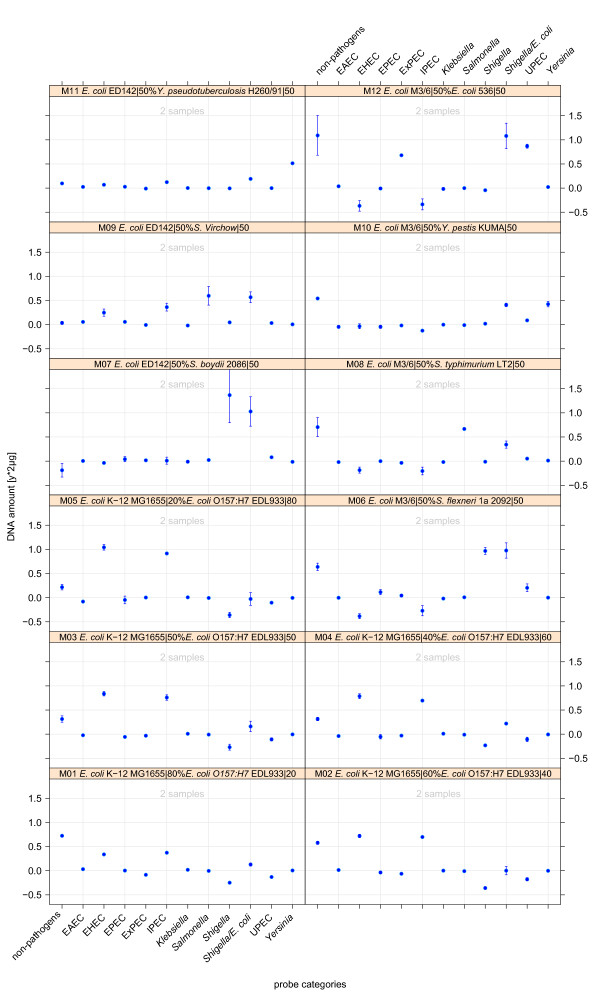
**Regression model behaviour on the categorical prediction of hybridisation patterns from new pathotypes that are not represented by specifically designed oligonucleotides**. The model training was based on the core pathotypes. The unspecific representation resulted in diffuse prediction outcome, where only the group of enteroinvasive *E. coli *shows cross-reactions to probes of *Shigella *and intestinal pathogens.

Moreover, we conducted test hybridisations with genomic DNA from different *E. coli *pathotypes (ETEC, EIEC, and SEPEC) without specific representation on the microarray. Thus, the tests could be considered as a kind of negative test with respect to the pathotypes in focus. With respect to level equivalence, patterns of these pathogroups were set in contrast to other *E. coli *pathotypes. The predictions graphically displayed in Figure 6 did not reveal a clear tendency to any of the main pathotypes. Only the hybridisation patterns of EIEC isolates indicated some hybridisation to probes of intestinal pathotypes and *Shigella *isolates. The observed interrelation between *Shigella *and EIEC classes coincides with the high similarity of enteroinvasive *E. coli *and *Shigella *isolates concerning pathogenicity and genotype. ETEC and SEPEC hybridisation patterns did not fit to any core pathotypes, a result that correlates well with prior expectation.

### Classification of mixed bacterial cultures

**Figure 7:**
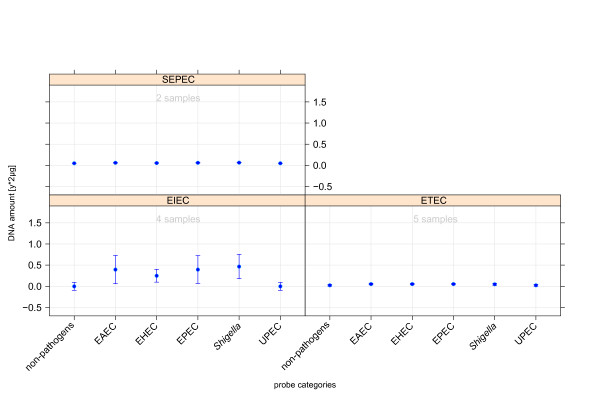
**Regression model behaviour on the categorical prediction of various mixed hybridisations**. The regression model was trained with pure sample and the mixed-culture hybridisation patterns (excluding new pathotypes like ETEC, EIEC and SEPEC).

Furthermore, the regression model was trained by specifically designed spike-in experiments to detect different pathotypes within mixed bacterial cultures. To maintain generality, hybridisation patterns of mixed culture samples did not serve as training data for the regression model. However, the predictions shown in Figure 7 did not only correlate with the true nature of test strains but also correctly quantified the underlying proportions. Especially the spike-in series with counter-rotated proportions of a non-pathogenic *E. coli *and an EHEC strain (Plots M01-M05) demonstrated the sensitivity of the regression model in estimations of quantities of bacterial DNA and its mixtures. Mixed culture test hybridisations did not reveal any limit of detectable rates of pathogens though it definitely exists. If such a limit is under-run - a possible scenario for faecal sample diagnostics - appropriate measures have to be taken to scale up group-specific DNA ratios in question.

### Antimicrobial resistance screening

The developed diagnostic microarray comprised features to screen for basic antimicrobial resistance (AMR) patterns in enterobacterial samples and communities. A set of 30 previously published AMR markers [14] was extended by 12 newly designed probes in order to extend the marker spectrum by probes for important AMR-classes like macrolides, but also by new variants of resistance genes of tetracyclines and β-lactams. The AMR probe set comprised genes coding for resistance-mediating enzymes and efflux pumps against aminoglycosides, β-lactams, sulfonamides, tetracyclines, dihydrofolate reductase (Dhfr) inhibitors, amphenicols and macrolides.

**Figure 8:**
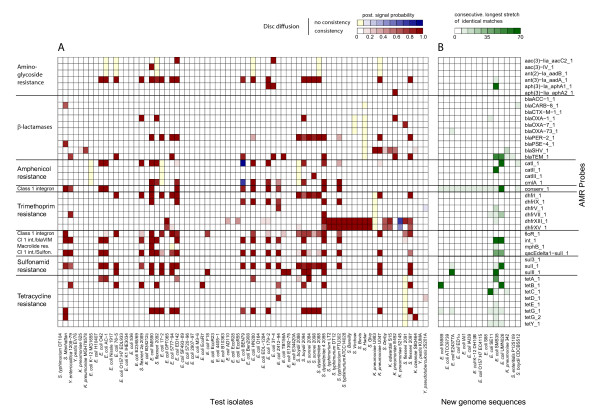
**Experimental antimicrobial resistance (AMR) screening among test isolates (A) and theoretical assessment of AMR in isolates with recently published genome (B)**. Colours in the left plot indicate posterior probabilities of membership to the signal fraction of the overall distribution of AMR-related hybridisation intensities. Additionally, the validation of disc diffusion tests is mapped on the microarray results. In case of consistency between microarray and disc diffusion results, the red colour scale is used, while the blue scale indicates no consistency. Yellow bars signify resistance in disc diffusion which was not detected by microarray analysis. Test hybridisations of pure culture DNA are plotted against AMR probes (right axis labels) while the type of AMR is provided as axis labels on the right. Colours of matrix B indicate the length of longest consecutive matches obtained by Smith-Waterman alignments of AMR probes against genome sequences.

AMR relevant, log normalised signal intensities of hybridisation patterns from all test strains were classified into a signal and a noise fraction by fitting a Gaussian mixture model composed of two normal distributions on all data points. Figure 8A summarises single posterior signal probabilities of AMR probe intensities obtained from numerous test hybridisations (levels in respective colour gradients). For about one fourth of hybridisation profiles, mainly originating from *E. coli *and *Shigella *isolates, no resistance markers could be detected. All tested *Salmonella *strains exhibited hybridisation signals indicative of resistance to trimethoprime (genes *dhfrXIII *and *dhfrXV*, exception *S. *Manhattan), whereas the hybridisation patterns of none of these isolates revealed any indication of resistance to sulfonamides. These two therapeutics are frequently applied in combination. A third fraction of strains was predicted to exhibit multiple antibiotic resistances. Genes coding for SHV-type (sulfhydryl variable) β-lactamases were in correspondence with a previous report only detected in *Klebsiella *isolates [26].

Microarray results were validated by susceptibility tests based on the disc diffusion method. Randomly verified negative results (no detected AMR) especially in the laboratory K-12 strain MG1655 completed the evaluation. Figure 8A reflects the mapping of results from disc diffusion assays to posterior signal probabilities of microarray hybridisations. The red colour scale reports agreement of microarrays and disc diffusion results, while the blue colour scale indicates the detection of resistance only by the microarray. Yellow bars indicate detected resistance by disc diffusion though the hybridisation pattern did not reveal any signal in the respective AMR class.

In almost all tests, the disc diffusion method confirmed the antibiotic resistances predicted by the microarray analysis. The laboratory *E. coli *K-12 strain MG1655 served as a control in AMR experiments. The *E. coli *K-12 genome contains the AMR genes *ampC*, *macAB*, *emrAB *and *acrAB*. AmpC functions as a penicillinase which especially affects ampicillin and other penicillins and therefore mediates resistance to oxacillin and amoxicillin. MacAB, EmrAB and AcrAB form efflux proteins in the extracellular matrix, which are specialised transporters of macrolides and provide erythromycin resistance [27,28]. As these protein complexes constitute common chromosomally encoded AMR structures, the respective genes were not considered in the described design of an AMR diagnostic. The disc diffusion experiments further revealed widespread susceptibilities to ceftriaxone. Resistance to third-generation cephalosporines mainly arises from the CTX-class (cefotaxime) of β-lactamases, and the hybridisation experiments did not exhibit any positive signals for the corresponding probe. Sporadic ceftriaxone resistances can be traced back to certain oxacillinases (*bla*OXA) or to PER-type (*Pseudomonas *extended resistance) extended-spectrum β-lactamases (ESBLs) [29].

### Designed probes and recently published enterobacterial genomes

Since the start of the probe selection, several new enterobacterial genomes have been published. They contain novel sequence information, a knowledge that impacts strain typing and diagnostics in general. This knowledge, especially of strains from new pathotypes, could, however, not be integrated in the developed microarray. Nevertheless, the microarray's diagnostic accuracy on these strains was assessed by Smith-Waterman alignments of all probe sequences against the genome sequences specified in part B of Table 1.

### Typing of pathogroups

**Figure 9:**
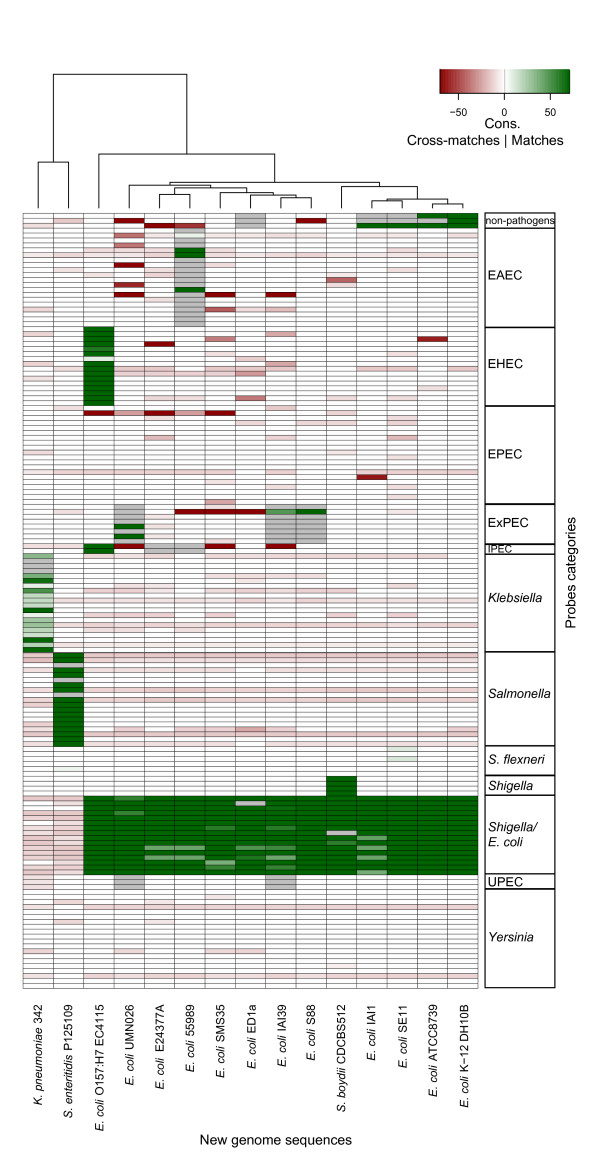
**Theoretical assessment of diagnostic probe performance based on alignments of probe sequences to recently published enterobacterial genomes**. The image plot summarises lengths of longest stretches of consecutive matching (green scale) and cross-matches (red scale) of probes to new genomes. Grey colour indicates an expectation of matching without the observation of matches. Fields coloured in light red represent weak similarities that will not lead to cross-hybridisation. The genus level categories show high similarity to corresponding probes, in downstream levels few cross-matching was observed between *E. coli *pathotypes. The cross-matching goes back to only few probes.

The updated data regarding recently published enterobacterial genome sequences mainly comprised non-pathogenic *E. coli *strains as well as UPEC, MNEC, EHEC, ETEC, EAEC and *Shigella *pathogroups but also *S. Enteritidis *and *K. pneumoniae *strains (Table 1). Among these, the MNEC, and ETEC pathogroups are not represented by specific capture probes (except for probes of the umbrella groups '*Shigella E. coli*', 'ExPEC' and 'IPEC'). The alignment results were summarised in Figure 9 as an image plot of strains against pathogroups. The plot indicates a correspondence of matching category and true pathogroup (green scale), no matching though it was expected (grey colour) or cross-matching (red scale). Colour intensities refer to the length of the respective longest consecutive stretch of matches.

The ability of genus level capture probes to discriminate between '*Shigella*/*E. coli*' and '*Salmonella*' isolates was confirmed by the alignments. The *K. pneumoniae *342 genome shows sequence similarity to almost all '*Klebsiella*'-specific capture probes. Moreover, the strain's unambiguous detection was further supported by the absence of sequences with similarity to probes from other pathogroups. According to the alignments, *Salmonella *and non-pathogenic *E. coli *strains could be clearly typed based on the probes included on the microarray although their genome sequences did not cover the full set of capture probes designed for these groups. Representatives of *E. coli *pathogroups which have not been included into the initial genome-wide probe selection (ETEC: *E. coli *strain E24377A; MNEC: *E. coli *strain S88) could be correctly classified as *E. coli *isolates and their genome sequences did not reveal a substantial risk of cross-hybridisation. Additionally, the genomes of UPEC strains IAI30 and UMN026 exhibited theoretical hybridisation patterns characteristic for the ExPEC pathogroup. Single cross-matching behaviour could be balanced by subsequent regression on the full set of group-specific capture probes.

### Assessment of AMR detection

In addition, the AMR-associated probe set was evaluated by screening for sequence similarities in recently published complete genome sequences of phenotypically characterised strains. Figure 8B provides lengths of the longest consecutive matches encoded in a green colour gradient. The SECEC strain SMS-3-5 was reported to be resistant against multiple antibiotics [30]. This finding could be confirmed by our sequence alignments which uncovered resistance loci coding for a TEM-type β-lactamase (*bla*TEM), a chloramphenicol acetyltransferase II (*cat*II), an aminoglycoside 3'-phosphotransferase (*aph*(3)-Ia *aphA*1), a tetracycline efflux protein (*tetA*) and a type II sulfonamide resistant dihydropteroate synthase (*sul*II). Genome sequence analysis indicated a second multiple resistant strain, i.e. UPEC isolate UMN026. The strain's genome encodes in correspondence to probe alignments for the TEM-type β-lactamase, the aminoglycoside/multi-drug efflux protein AcrD, the dihydropteroate synthase type-1 and several efflux pumps. Resistances to single antibiotics were also indicated by sequence alignment of the AMR-related probe set to the genomes. *E. coli *strains 55989 and SE11 were predicted to be tetracycline-resistant whereas strain E24377A was shown to carry determinants for resistance against sulfonamides. Although our AMR-related probes did only reveal moderate similarity to three different regions in the *K. pneumoniae *isolate 342, the strain was described to be highly resistant. The resistance mechanisms in *K. pneumoniae *342 rely on β-lactamases and on the existence of many efflux pumps [31]. The β-lactam resistance was detected via sequence alignment of the *K. pneumoniae *342 genome with the designed probes.

### Overview of classification results

In summary, the classification of DNA hybridisation based on signal intensities of specifically designed markers of enterobacterial pathogroups yielded accurate results throughout all levels of hierarchical diagnostic decisions. The prediction outcome was stable regarding different compositions in training sets of the regression model and regarding contrasts between groups from different pathogroup levels. Overall, the regression model exhibited low levels of prediction noise in non-target classes. Accuracies in predictions of the amount of hybridised DNA depended on the number of biological repeats, the distinction power and amount of group-specific probes and the homogeneity of the pathogroup in focus. Spike-in experiments of mixed cultures underlined the ability of the diagnostic microarray in conjunction with regression analysis to decode the proportions of bacteria in clinical specimens. The microarray proved to detect major AMR conferred by degrading enzymes or efflux proteins and the established signal analysis provided information on the reliability of resistance prediction as posterior probabilities. Further screening of newly published enterobacterial genomes regarding the occurrence of the designed capture probes confirmed their basic validity.

## Discussion

We present a novel, complete strategy concerning design and analysis of a diagnostic microarray for the distinction of subgroups within the versatile family of *Enterobacteriaceae*. Members of this family are known as commensals but also as versatile pathogens. The multiplicity in clinical symptoms implies a large gene pool, genetic exchange and the requirement of complex diagnostic tests [1,3,5,32,33].

The DNA microarray platform provides a suitable high-throughput environment to determine a large number of traits within a single diagnostic test. The diagnostic strategy applied here was based on an initial categorisation of the target group of bacteria. The subsequent probe selection was geared to the prior categorisation and its quality and discrimination power certainly depends on a proper choice of meaningful sub-entities in the reference set of target genomes. As an example, non-pathogenic *E. coli *strains form an inhomogeneous and insufficiently characterised subgroup. Beside commensal intestinal isolates, the subgroup was composed of e.g. laboratory strains like the K-12 isolates but also included the strain Nissle 1917, which genotypically resembles ExPEC strains without expressing ExPEC-specific virulence factors [34] and the commensal isolate HS [35]. *E. coli *K-12 derivatives are in use as laboratory strains for nearly 90 years and were frequently passaged and genetically manipulated. Therefore the K-12 lineage does not represent 'typical' commensals. Though this heterogeneous subgroup could be characterised by oligonucleotide determinants, it would be advantageous in a diagnostic context to focus on many 'true' commensals that were isolated from the intestinal tract, given the respective genomic data. These examples underline the importance of well defined bacterial subgroups in order to enhance the performance of any microbial diagnostic device.

The initial search algorithm of probe selection, longest common factor statistics, explicitly scans the whole genomes with coding and non-coding regions. The consideration of non-coding areas as robust markers with respect to specify a group of bacteria is not straight-forward. Non-coding regions are expected to be largely less conserved. Nevertheless, highly conserved intergenic motifs like repetitive sequences termed ERIC (enterobacterial repetitive intergenic consensus) [36] or conserved transcriptional regulatory elements [37] were described for enterobacteria previously. Our study confirms by the high number of selected intergenic probes, which are distributed to nearly all levels of considered clinically relevant subgroups, the existence of characteristic traits outside of coding regions. The fact that non-coding regions could have regulatory functions is well known and DNA sequence alterations in such regions may thus affect a broad variety of bacterial traits including physiology, but the knowledge of the concrete DNA regions and their functionality is still poor. The detected conserved diagnostic markers will provide a starting point for further research on the impact of these DNA regions.

### Microarray-based diagnostics in comparison

The microarray technology is well suited for diagnostic applications due to its highly parallel architecture. In the past few years many workgroups studied the applicability of microarrays to microbial ecology and phylogenetics [38,39], comparative genomics [40-42] and clinical diagnostics [15,43]. Microbial diagnostic microarrays (MDM) are generally characterised by a low number of probes, which either target sequence differences in single diagnostic markers or represent a library of virulence-associated genes. MDM from the first category rely on probes designed from sequence differences in single markers like 16S rRNA [44] and *gyrB *[11]. Though these single marker diagnostics perform well in the distinction between distantly related organisms, its distinction performance on subspecies level was found to be limited [45]. Other MDM were based on libraries of determinants for known virulence-associated genes [13,14,46,47]. The extension of such probe sets by so far uncharacterised genomic regions has been shown to improve the discrimination of closely related bacterial variants [48]. However, high rates of horizontal gene transfer and recombination, which frequently occur especially among *E. coli *and other enterobacteria [49], can also affect virulence-associated genes due to selection pressure in the host. Furthermore, virulence determinants are often associated with pathogenicity islands, and are subjected to frequent alterations due to genome plasticity [32,50]. Extraintestinal pathogenic *E. coli *isolated from different human and animal hosts have largely congruent virulence-associated genome contents and the overall genome content of many non-pathogenic *E. coli *isolates resembles that of extraintestinal pathogenic isolates. Thus, the detection of known virulence-associated genes does not allow proper strain typing and risk assessment [34,51-54]. Consequently, the proposed strategy in the development of a MDM benefits from the determination of the genome-wide most stable subgroup-specific traits among available non-redundant genomic information of the target group of bacteria.

### AMR screening

An important part in clinical treatment of bacterial infections is the choice of an appropriate drug therapy. In this context, the integration of a screening for important determinants of antimicrobial resistances was mandatory in the development of a diagnostic tool. The AMR screening feature does not only provide an assessment of appropriate antimicrobial resistance determinants, but also enables the tracking of AMR progression. Our developed screening based on probes for the major classes of AMR mediated by enzymes or efflux proteins extends previous studies [14] in complexity and analysis methodology. By fitting a Gaussian mixture model to AMR-related signal intensities, we provide an indication of the reliability of microarray signals. Hybridisation with a large number of test strains and *in vitro *verification of resistances by the disc diffusion method largely correlated in our study. Exhaustive AMR analysis would require a wealth of capture probes to track all potentially occurring single nucleotide polymorphisms and is out-of-scope for high-throughput pathogen diagnostics.

The challenge to establish microarray-based diagnostics of AMR with differences between microarray detection and conventional testing was already stated in previous studies [16]. As *E. coli *strains possess a high number of drug efflux systems and an even higher number of other membrane transporters [55], a functional shift mediated by mutations could be the cause for such observed differences. Nevertheless, microarray-based detection of AMR has been described previously as a support of conventional susceptibility testing [14,16]. Here, microarrays were successfully applied to survey the occurrence of different classes of AMR in a wide range of enterobacterial isolates.

### Diagnostics of enterobacteria

The microarray design strategy was optimised for the classification of clinically relevant enterobacteria. Probe selection was based on a previously published longest common factor approach and on subsequent filtering of candidate capture probes according to strict match and mismatch limits, which conferred robust signalling with low cross-hybridisation. By 'unsupervised' evaluation of all possible subgroupings with distinct oligonucleotide patterns, the ability to distinguish *S. flexneri *from other *Shigella *species underlines the high sensitivity in strain typing mediated by the applied probe selection strategy. Extensive test hybridisations were conducted in order to assess the quality of the selected probe set and to obtain training data for the calibration of the regression model.

Probe-wise performance evaluations based on these tests legitimate the separation of sense and antisense capture probes, which exhibited divergence of support quality e. g. in classifications of *Yersinia *spp. test isolates. Detailed investigation concerning the nature of the selected probes revealed single markers, which were previously described as group specific. As an example two capture probes of the EAEC pathogroup indicating strong group-specific support are derived from the *aat *gene locus. The whole *aat *and *aap *loci were previously reported to be specific for EAEC strains and suggested for diagnostic purposes [56]. The classification of *Shigella *isolates is mainly conferred by capture probes derived from the *ipaH *gene. Venkatesan and co-workers [57] already described motifs of this gene locus to be effective markers of *Shigella *and EIEC virulence. Future availability of EIEC genomes will enable robust design of joint capture probes for the invasive pathotype. The function of nearly half of the capture probes is still uncharacterised and to our knowledge these markers were not applied in enterobacterial diagnostics before. The finding underlines the importance of an unsupervised probe selection mechanism considering both coding and non-coding genomic regions.

Test strains were classified to enterobacterial subgroups by a regression model. The model was able to provide clear separation of the considered subgroups while the prediction accuracy of nature and amount of hybridised DNA increased with the size of the training set and the distance between the groups. Spike-in experiments with mixed culture hybridisations containing isolates from two groups in various proportions were intended to evaluate the power of classification for bacterial communities. The tendencies of predictions based on these mixed culture hybridisations were mainly correct. The regression model is generally able to determine the composition of bacterial communities. By conducting comprehensive tests with biological repeats, the prediction performance of the regression model can certainly be further improved as shown for pure culture predictions. In extremely unbalanced mixtures, especially if single strains are highly underrepresented, the implementation of an amplification technology may circumvent the existence of detection limits [58].

In a separate *in silico *analysis we matched the probe set to recently published enterobacterial genomes. The assessment of probe validity on yet unconsidered sequence information confirmed the appropriateness of selected probes. Major AMR patterns reported for these strains could be recognised by the corresponding capture probes of the developed microarray thus recommending it for AMR diagnostics.

Regarding the numerous existing approaches to construct a diagnostic tool for *Enterobacteriaceae *or its subgroup *E. coli*, our design strategy differs because of its genome-wide probe selection, the broad range of targets and an intuitive but powerful regression model for the analysis of hybridisation patterns. As a proof-of-principle, the probe selection was based on genomic data of published strains that represent clinically relevant phenotypes. With an increase in genomic data, the method of probe selection will even gain in accuracy of detecting stable traits of the bacterial groups in focus. The chosen microarray platform with twelve separate spotting areas provides a tool for highly parallel diagnostics to reduce analysis time and costs. The trade-off is a limited number of probes, but the obtained test results proved the suitability of the probe set selected for the distinction of the assigned clinical phenotypes. Further efforts should be focused on the reduction of costs for a single hybridisation. A recently developed label-free system might be a step in the right direction as it reduces the preparation and hybridisation time of samples and in parallel increases the sensitivity [59].

## Conclusions

The basic concept and analytical elements of the described microarray development can be easily transferred to other bacterial taxa and even beyond. Although the microarray design was focused on clinical diagnostics, its application to further fields like quality control of food or water as well as veterinary medicine is imaginable. In summary, a novel, complete developmental process of a diagnostic microarray, which enhances the diagnostic reliability, especially on subspecies levels, is described. The specifically adapted regression model further improves the diagnostic performance via continuous learning abilities in the process of its application.

## Methods

### Probe selection, sequence alignments and functional annotation

Rahmann [60] proposed an algorithm based on enhanced suffix arrays to identify all common, contiguous subsequences, termed factors, in a subset of reference genomes (see part A of Table 1). The method is based on the definition of appropriate matching (here *l *= 70 bases) and cross-hybridisation (*c *≤14 bases) thresholds to ensure a safe matching to all target sequences within genomes of a group and to prevent for undesired matches to any areas in other genomes. Briefly summarised, the algorithm decomposes the target genomes into all possible factors and selects those subsequences of the chosen length *l *= 70 bases that uniquely occur in each genome. To deal with multiple genomes, a joint suffix array of multiple genomes was generated, which enables the efficient extraction of common subsequences. Potential diagnostic groups are defined in an unsupervised manner by the existence of a set of common factors with a minimum length *l *and maximum common length *c *to any factors of other genomes. Matching statistics and longest common factors were obtained according to the algorithm described by Rahmann [61].

The set of probe candidates was further investigated according to compositional complexity, GC-content, change in Gibb's free energy upon hybridisation, melting temperature and cross-hybridisation to human DNA. Reverse complementary oligonucleotides were considered as autonomous candidate probes even if they fully overlap, as the difference in base composition may have an influence in hybridisation properties. All alignments of selected probes to recently published genome sequences or the human genome were carried out using the software PARALIGN [62] in Smith-Waterman mode. PARALIGN was also applied to align candidate probes with the human genome to evaluate cross-hybridisation risk in clinical specimens. Similarly, the performance of the probe set was assessed on recently published enterobacterial genomes.

All oligonucleotides related to pathogroup typing were functionally annotated by homology-based knowledge transfer using the NCBI-BLAST search and the enterobacterial sequence database. Annotations were obtained manually from the most abundant function assigned to respective genomic regions. New AMR-specific capture probes were designed by the programme OligoPicker [63]. Probe uniqueness was validated against the gDNA of reference strains with BLAST [64].

### Test hybridisations

Samples of gDNA extracted from representative strains of various enterobacterial pathogroups were hybridised to the microarray in order to determine its classification performance. The test set is composed of 40 *E. coli *isolates subdivided to two MNEC, two SEPEC, three UPEC, five EHEC, four EAEC, 4 EIEC, six non-pathogenic *E. coli*, three APEC (avian pathogenic *E. coli*), five EPEC and six ETEC. Furthermore the set contains 17 *Shigella *from species *S. dysenteriae *(two), *S. sonnei *(two), *S. flexneri *(eight) and *S. boydii *(four), 16 *Salmonella *with seven *S. *Typhimurium and several other serovars therein, 13 *Klebsiella *consisting of 11 *K. pneumoniae *and the species *K. ozeanae *and *K. edwardsii *as well as six *Yersinia *spp. strains representing two *Y. pestis*, *Y. pseutotuberculosis *and *Y. enterocolitica *isolates. Further details on the identity of selected isolates are given in Table S3.3 of additional file 3.

Faecal samples as well as many clinical specimens are composed of mixed bacterial communities comprising pathogens and non-pathogens. The evaluation of the microarray accounts for these types of clinical diagnostics by specifically designed spike-in experiments. The experiments target evaluations with respect to the contrasting ability of the microarray in the background of multiple bacteria and the sensitivity in determining proportions of their occurrence in clinical samples.

### Preparation of genomic DNA

Cultures were grown overnight at 37°C in LB (Luria broth). Genomic DNA was isolated following standard protocols [65].

### Microarray technology and hybridisation scheme

The HTA Slide12^™ ^from Greiner Bio-One provides 12 separate wells for independent parallel hybridisation. They are composed of polymer and coated with a 3D-epoxy surface. Each well provides a printable area of 12 × 36 mm² bordered by a rim of 0.5 mm in height. The 70-mer oligonucleotides were synthesised by metabion international AG (Martinsried, Germany) and spotting of microarrays was conducted with a spot distance of 225 μm by Scienion AG (Berlin, Germany) using a sciFLEXARRAYER S11 piezo dispenser.

Test hybridisations with different combinations and ratios of mixed culture samples were set up in addition to pure culture tests in order to evaluate the performance of the microarray on community samples (see also Additional file 3, Table S3.1). The experiments comprised a dilution series of a mixture of non-pathogenic *E. coli *K-12 strain MG1655 and the EHEC O157:H7 isolate EDL933 (Figure 8, M01-M05). The spike-in experiments were intended to evaluate the accuracy to predict simultaneously the DNA content and therefore the amount of two or more bacterial groups in a test sample. For the spike-in mixtures of the K-12 and the EHEC strain, the pathogroup-specific rates varied in a range between 0.8 and 0.2 of overall hybridised DNA in a counter-rotated mode starting with an amount of 1.6 g (ratio 0.8) K-12 DNA in plot M01. The applied regression model was trained with all hybridisation patterns of groups indicated as annotation of the x-axis in the plots. To calibrate the coefficient matrix for the prediction of mixed cultures, the training was extended by the mixed-culture patterns. All mixed culture experiments were conducted with a technical repeat. In these cases no cross-validation was performed.

### Probe labelling and array hybridisation

Genomic DNA was labelled with the DecaLabel DNA Labeling Kit from Fermentas (St. Leon-Rot, Germany) and 1 mM Cy5-dUTP dye (Enzo Life Sciences, USA). The MinElute PCR purification kit from Qiagen (Hilden, Germany) was used for gDNA purification. All solutions applied in processing and washing procedures of the slides were demineralised and filtrated with 0.22 μm pore filters. Slides were treated for 5 min under agitation with 0.1% Triton X-100. Afterwards, they were transferred twice to a processing chamber filled with 6 mM HCl and agitated for 2 min. After that, they were placed under agitation in 100 mM KCl solution for 10 min and then in water for 2 min. Then, slides were transferred to a chamber filled with pre-warmed (50°C) 50 mM ethanolamine, 0.1% sodium dodecyl sulfate (SDS) in 0.1 M Tris (pH 9.0) for 15 min. Processing was completed by two washing steps with ultra-pure water for 2 min under agitation, rinsing in cold ethanol and drying for 3 min under centrifugation at 1,000 g.

Prior to hybridisation, 2 μg of labelled and purified samples were dried in a speedvac and resuspended in 15 μl hybridisation buffer (Scienion SciHyb, prewarmed for 10 min to 42°C). The cavities of the hybridisation chamber were loaded with 20 μl H_2_O, samples were dropped contactless on the spotted areas of the slides and the slides were hybridised overnight (about 15 h) in a 42°C water basin.

After removal of hybridisation fluid, the arrays were washed three times with 30 μl washing solution 1 (5% 20 × sodium chloride-sodium citrate (SSC), 0.033% SDS). The slides were then consecutively transferred to chambers with washing solution 1, 2 (1% 20 × SSC) and 3 (0.25% 20 × SSC) and agitated for 5 min each. Finally, the slides were dried by centrifugation at 1,000 g for 3 min.

The slides were scanned in 5 μm resolution with an Axon GenePix 4000B microarray scanner (MDS Analytical Technologies, Ismaning, Germany). Scan images were processed by applying the GenePix 6.0 software to obtain raw intensities.

### Disc diffusion test

Strains with predicted antibiotic resistances based on the microarray hybridisation were cultivated overnight at 37°C in Mueller-Hinton (MH) medium. 100 μl of the overnight culture were transferred to 4 ml MH medium and cultivated for 4 h under constant shaking at 37°C. These cultures were diluted to a final cell count of 1 × 10^6 ^- 5 × 10^6 ^colony forming units/ml. 100 μl of each dilution were plated on a MH-agar plate. Discs containing the antibiotics listed in Additional file 3, Table S3.2 were placed on the agar plates which were then incubated overnight at 37°C. The assignment of susceptibility, intermediate behaviour or resistance was subsequently determined by measuring the diameter of the growth inhibition zone around the susceptibility discs (see Additional file 3, Table S3.2 for reference values). According to the large spectrum of different antimicrobial agents [29], the class of β-lactamases was represented in the experiments by aminopenicillin, isoxazolylpenicillin and cephalosporin subclasses.

### Evaluation of hybridisation patterns

Subsequent microarray analyses were performed using the statistical programming software R [66].

### Normalisation and processing of AMR signal intensities

Raw intensities were normalised by the algorithm for variance stabilisation between arrays [67]. The method homogenises the variance of hybridisation intensities from a set of samples by transformation of the data with the model *h*(*x*) = arcsinh (*a *+ *bx*). This transformation, applied as R implementation vsn, corrects for an underweighting of differences in lower intensities.

Microarray experiments yield two kinds of outcomes: the signal intensities upon binding of complementary DNA and an unspecific fluorescence of the microarray surface or dye remnants. For log normalised hybridisation patterns each type of intensity values follows a normal distribution. The classification accuracy of microarray intensities in either one of these classes is strongly dependent on the degree of overlap of the two distributions. In experimentally generated hybridisation patterns the bimodal Gaussian mixture model is able to fit the two intrinsic normal distributions. Parameter estimation of the Gaussian mixture and calculation of posterior probabilities of the classification was achieved by using the R-package Mclust [68].

### Analysis of variance and simultaneous inference of multiple comparisons

An ANOVA was applied to determine the general potential of capture probes concerning the identification of differences in signal intensities across contrasted bacterial groups. By fitting an aov model, the R implementation of ANOVA in the stats-package, the probe-wise occurrence of distributional differences of signal intensities in any bacterial group and for all capture probes was tested.

In case of a detected difference in mean signal intensities of a capture probe between the target and non-target pathogroups, additional tests like the Tukey honestly significant difference are often applied in such a context. In the described analysis we applied the simultaneous inference of one-sided multiple comparisons [21]. The algorithm evaluates individual test hypothesis derived from an ANOVA model as max-t type test statistics. In terms of microarray intensity data the method was applied to compare individual differences based on ANOVA model parameters between all pairs of bacterial groups under a global error model. The method is implemented in the R-package multcomp [69] and yields adjusted p-values.

## Abbreviations

IPEC: intestinal pathogenic *E. coli*; EHEC: enterohaemorrhagic *E. coli*; EPEC: enteropathogenic *E. coli*; ETEC: enterotoxigenic *E. coli*; EAEC: enteroaggregative *E. coli*; EIEC: enteroinvasive *E. coli*; ExPEC: extraintestinal pathogenic *E. coli*; UPEC: uropathogenic *E. coli*; MNEC: Meningitis-associated *E. coli*; SEPEC: Sepsis-associated *E .coli*; AMR: antimicrobial resistance; ANOVA: analysis of variance; gDNA: genomic DNA; ESBLs: extended-spectrum β-lactamases; MDM: microbial diagnostic microarray; APEC: avian pathogenic *E. coli*;

## Authors' contributions

UD and TM designed research together with SR, TD and JH. SR developed and adapted the longest common factor based probe preselection algorithm. TF performed probe selection, microarray experiments and data analysis. WW produced the microarray and supported the microarray experiments. ER, FG, WR and AF provided bacterial strains. TF wrote the manuscript assisted by UD, TM, SR, TD and JH. All authors have read and approved the final manuscript.

## Supplementary Material

Additional file 1 **Supplement1.txt**. Additional file 1 contains a list that maps internally used probe identifiers with identifiers used in the NCBI probe database. Click here for file

Additional file 2 **Supplement2.doc**. Additional file 2 contains information to probe performance and classification of the intermediate pathogroup level. Click here for file

Additional file 3 **Supplement3.doc**. Additional file 3 contains supplementary information of the composition of mixed culture test samples and the standards of susceptibility assignments for the tested antimicrobial agents. Click here for file
