# Supplementary material for: High-throughput microarray technology in diagnostics of enterobacteria based on genome-wide probe selection and regression analysis
Source: BMC Genomics. 2010 Oct 21;11:591. doi: 10.1186/1471-2164-11-591 (PMC3017858; doi:10.1186/1471-2164-11-591)
Supplement: Additional file 2 — Supplement2.doc. Additional file 2 contains information to probe performance and classification of the intermediate pathogroup level. [file 1471-2164-11-591-S2.DOC]

**Classifications across the intermediate pathogroup level**

In general, higher p-values of capture probe performance were obtained in the intermediate level downstream of the *E. coli* group mainly because of closer evolutionary distances between these groups compared to the distances in the genus level. Figure 3 depicts probe performance evaluations among the pathogroups denoted ‘*Shigella*’, ‘non-pathogens’, ‘IPEC’ and ‘ExPEC’.

The classification accuracy among the branch of *Shigella* and *E. coli* strains was evaluated in the intermediate level of the pathogroup tree (pathogroups shaded in blue in Figure 1). Figure S2.2 depicts the prediction results subdivided according to classes on this level. The training of the regression model was restricted to intensity data from probes designed for respective pathogroups of the intermediate level. Even in the narrow evolutionary spectrum of *E. coli* isolates the regression model was able to safely separate hybridisation patterns of *Shigella*, ExPEC and intestinal strains. Again, the level of prediction noise in non-target pathogroups was basically absent except for a reciprocal interference between non-pathogenic and IPEC. As described in the main document, this interference could be resolved in predictions contrasting the non-pathogenic pathogroup against *E. coli* pathotypes (see Figure 6).

##
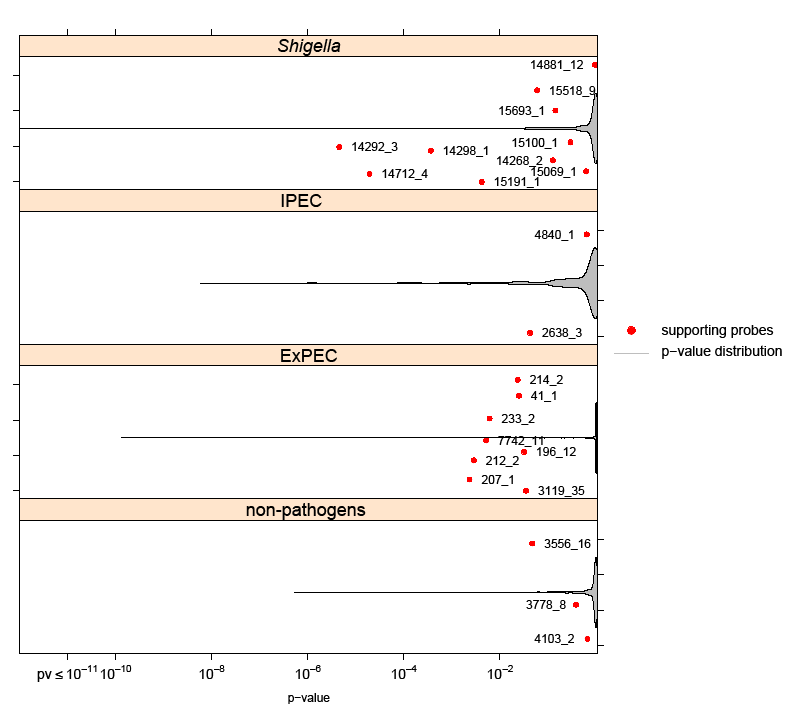
Figure S2.1 - Evaluation of single probe support within the intermediate level of the pathogroup tree.

The significance of discrimination power of single group-specific probes was determined as described above. The minor support in six *Shigella* probes arises from its specificity to the *S. flexneri* subgroup of *Shigella*. The generally lower p-values in comparison to the genus level indicate putatively results from closer relationships of the groups and therefore a smaller genetic variability.

##
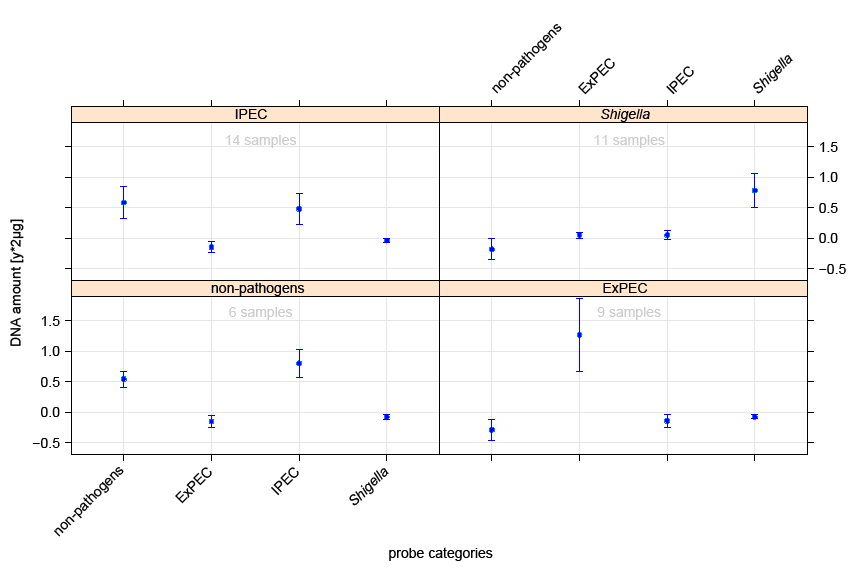


## Figure S2.2 - Classification of hybridised DNA of the intermediate level of pathogroups beneath the node of *E. coli* and *Shigella* isolates.

Figure S2.2 contrasts pathogroups of the intermediate *E. coli* level with ExPEC and IPEC categories and further contrasts to *Shigella* and non-pathogenic *E. coli*.
