# Supplementary material for: High-throughput microarray technology in diagnostics of enterobacteria based on genome-wide probe selection and regression analysis
Source: BMC Genomics. 2010 Oct 21;11:591. doi: 10.1186/1471-2164-11-591 (PMC3017858; doi:10.1186/1471-2164-11-591)
Supplement: Additional file 3 — Supplement3.doc. Additional file 3 contains supplementary information of the composition of mixed culture test samples and the standards of susceptibility assignments for the tested antimicrobial agents. [file 1471-2164-11-591-S3.DOC]

## Table S3.1: Table of mixed culture tests of microarray hybridisations.

| gDNA 1 | Ratio | gDNA 2 | ratio |
| --- | --- | --- | --- |
| *E. coli* K-12 MG1655 | 0.8 | *E. coli* O157:H7 EDL933 | 0.2 |
| *E. coli* K-12 MG1655 | 0.6 | *E. coli* O157:H7 EDL933 | 0.4 |
| *E. coli* K-12 MG1655 | 0.5 | *E. coli* O157:H7 EDL933 | 0.5 |
| *E. coli* K-12 MG1655 | 0.4 | *E. coli* O157:H7 EDL933 | 0.6 |
| *E. coli* K-12 MG1655 | 0.2 | *E. coli* O157:H7 EDL933 | 0.8 |
| *E. coli* M3/6 | 0.5 | *S. flexneri* 1a | 0.5 |
| *E. coli* ED142 | 0.5 | *S. boydii* 2094 | 0.5 |
| *E. coli* M3/6 | 0.5 | *S.* Typhimurium LT2 | 0.5 |
| *E. coli* ED142 | 0.5 | *S. Infantis* | 0.5 |
| *E. coli* M3/6 | 0.5 | *Y. pestis* KUMA | 0.5 |
| *E. coli* ED142 | 0.5 | *Y. pseudotuberculosis* H260/91 | 0.5 |
| *E. coli* M3/6 | 0.5 | *E. coli* 536 | 0.5 |

The table lists the compositions of test samples prepared for hybridisation experiments with mixed cultures. The first 5 spike-in experiments refer to the evaluation of detection accuracy in samples of varying gDNA amounts of a commensal against an EHEC strain.

## Table S3.2: Standards of antimicrobial resistance.

| Antibiotic | Class | Concentration[μg/ml] | Resistant  [mm] | Intermediate  [mm] | Susceptible  [mm] |
| --- | --- | --- | --- | --- | --- |
| Amoxicillin | β-Lactam (Aminopenicillin) | 2 | ≤ 15 | 16-22 | ≥ 23 |
| Oxacillin | β-Lactam (Isoxazolylpenicillin) | 5 | ≤ 15 | - | ≥ 16 |
| Ceftriaxone | β-Lactam (Cephalosporin) | 5 | ≤ 15 | - | > 15 |
| Gentamicin | Aminoglycoside | 10 | ≤ 14 | 15-20 | ≥ 21 |
| Erythromycin | Macrolide | 15 | ≤ 16 | 17-20 | ≥ 21 |
| Tetracyclin | Tetracyclin | 30 | ≤ 16 | 17-21 | ≥ 22 |
| Chloramphenicol | Amphenicols | 10 | ≤ 20 | - | ≥ 21 |
| Sulphomethoxazole/Trimethoprim | Sulfonamid/Dr inhibitor | 25 | < 15 | 15-17 | > 17 |

Standard values to assign susceptibility to antibiotics. The thresholds were defined by the Clinical and Laboratory Standards Institute (USA). The listed antimicrobial agents cover all classes for which equivalent resistance probes were designed.

## Table S3.3 - Table of isolates used in microarray tests

| Species | Patho-/ Serotype | Isolate |  |
| --- | --- | --- | --- |
| *E. coli* | MNEC | IHE3034 |  |
|  |  | A21 |  |
|  | SEPEC | 4405/1 |  |
|  |  | B10363 |  |
|  | UPEC | 536 |  |
|  |  | AD110 |  |
|  |  | ECOR55 |  |
|  | EHEC | ED142 |  |
|  |  | EDL933 |  |
|  |  | SF493/89 |  |
|  |  | 5720/96 |  |
|  |  | 2907/97 |  |
|  | EAEC | 5777/94 |  |
|  |  | O42 |  |
|  |  | 17-2 |  |
|  |  | DPT065 |  |
|  | EIEC | 76-5 |  |
|  |  | EDL-1284 |  |
|  |  | HN280 |  |
|  |  | O164 |  |
|  | non-pathogenic | ECOR28 |  |
|  |  | K-12 MG1655 |  |
|  |  | Nissle 1917 |  |
|  |  | M3/6 |  |
|  |  | ECOR7 |  |
|  |  | ECOR23 |  |
|  | APEC | BEN79 |  |
|  |  | BEN2908 |  |
|  |  | AC/I |  |
|  | EPEC | 179/2 |  |
|  |  | E2348/69 |  |
|  |  | 37-4 |  |
|  |  | Z412-94 |  |
|  |  | TB156A |  |
|  | ETEC | F18 |  |
|  |  | IMI590 |  |
|  |  | H10407 |  |
|  |  | E1392-75 |  |
|  |  | E34420A |  |
|  |  | B34212c |  |
| *S. dysenteriae* | 4 | 2095 |  |
|  | 9 | 2088 |  |
| *S. sonnei* | LT06 | 2084 |  |
|  | LT06 | 2083 |  |
|  | LT50 | 2098 |  |
| *S. flexneri* | 1a | 2092 |  |
|  | 2a | 2089 |  |
|  | 2b | 2090 |  |
|  | 3a | 2093 |  |
|  | 3a | 2082 |  |
|  | 3b | 2091 |  |
|  | 4a | 2081 |  |
|  | 5 | 2097 |  |
| *S. boydii* | 4 | 2087 |  |
|  | 4 | 2085 |  |
|  | 11 | 2086 |  |
|  | 14 | 2094 |  |
| *S.* Typhimurium | B | DT104 |  |
|  |  | DT17 |  |
|  |  | DT12 |  |
|  |  | DT170 |  |
|  |  | PTU302 |  |
|  |  | LT2 |  |
|  |  | ATCC14028 |  |
| *S.* Bareilly^1^ |  | 1138/98 |  |
| *S.* Infantis^1^ |  | 943/98 |  |
| *S.* Virchow^1^ |  | 318/98 |  |
| *S.* Livingstone^1^ |  | 868/98 |  |
| *S.* Bovismorbificans^1^ |  | 1222/98 |  |
| *S.* Manhattan^1^ |  | 913/98 |  |
| *S.* Hadar^1^ |  | 814/98 |  |
| *S.* Give^1^ |  | 1250/98 |  |
| *S.* Derby[[1]](#footnote-2) |  | 1329/98 |  |
| *K. pneumoniae* |  | MGH78578 |  |
|  |  | U983 |  |
|  |  | 375 |  |
|  |  | E492 |  |
|  |  | 625 |  |
|  |  | Bk098/2 |  |
|  |  | 3091 |  |
|  |  | Kp52145 |  |
|  |  | U047 |  |
|  |  | SB3464 |  |
|  |  | 110 |  |
| *K. ozeanae* |  | SB3431 |  |
| *K. edwardsii* |  | S15 |  |
| *Y. enterocolitica* |  | WA314 |  |
|  |  | 1208-79 |  |
| *Y. pseudotuberculosis* |  | 25201A |  |
|  |  | H260/91 |  |
| *Y. pestis* |  | KUMA |  |
|  |  | EV76 |  |

The marked *Salmonella* strains are all serovars of *S. enterica ssp. enterica*

The table lists all isolates applied for test hybridisations of the developed diagnostic chip. An abbreviated nomenclature was used in *Salmonella* listings providing genus and serovars.

## Figure S3.1 - Validation of antimicrobial resistance with the disc diffusion test.

## Resistances found by microarray hybridisations were tested in correspondence to the hybridisation results by the exposure of resistant strains to the following antimicrobial substances: Gentamicin (GM, Aminoglycoside), Ceftriaxone (CT, β-lactam), Oxacillin (OC, β-lactam), Amoxicillin (AC, β-lactam), Sulphometoxazole (SX, Sulfonamide), Tetracycline (TC), Trimethoprim (TP, DR inhibitor), Chloramphenicol (CP, Amphenicol) and Erythromycin (EM, Macrolide). Values specify the size of the zone of full inhibition, those in brackets the zone of partial inhibition. Dashes mark cases where no resistance was found in hybridisations and therefore no experimental validation was conducted. The colours are mapped according to the resulting categories from susceptibility to resistance. Most microarray-based resistance predictions could be confirmed by the experiments, though we also obtain susceptibilities in single strains. (^1^ The marked Salmonella strains are all serovars of S. enterica ssp. enterica)

1. [↑](#footnote-ref-2)
